# To Remove Nitrate of Silver Stains

**Published:** 1898-04

**Authors:** 


NITRATE of silver stains can be removed by rubbing with a cloth wet in one part each of corrosive sublimate and ammonium chloride to eight parts of water.

